# Rectal Ischemia Mimicked Tumor Mass

**DOI:** 10.1155/2013/853825

**Published:** 2013-09-12

**Authors:** Nicolaos Zikos, Panagiota Aggeli, Evangelia Louka, George Pappas-Gogos

**Affiliations:** ^1^Department of Surgery, Filiates General Hospital, 29 Kapodistriou, 1a Nizna, Filiates, 45332 Ioannina, Greece; ^2^Department of Gastroenterology, Filiates General Hospital, Eleftheriou & Kavafi, Filiates, 46100 Igoumenitsa, Greece; ^3^Department of Pathology, Filiates General Hospital, 99-101 G. Papandreou, N. Philadelphia, 14342 Athens, Greece

## Abstract

Ischemic proctitis is a rare disease which is usually encountered in elderly with comorbidities. We present a case of an 80-year old man with severe coronary disease who presented with severe hematochezia and hypotension. Endoscopy revealed a rectal mass 3-4 cm above the dental line and rectosigmoid mucosal inflammation compatible with ischemic colitis. The rectal insult was so intense that it resembled a neoplasmatic lesion. We discuss the causes, the prognostic factors, and the clinical and therapeutic challenges of this rare, albeit life-threatening entity, and we review the relative literature. A percentage of 10%–20% of patients with ischemic colitis usually have a distal potentially obstructing lesion or disorder such as cancer, diverticulitis or fecal impaction. Ischemic colitis, when mucosal and submucosal edema is severe and hemorrhagic nodules are large enough, can mimic a neoplasmatic lesion. The best treatment approach is a conservative management initially with a close clinical followup and after stabilization a repetition of rectal endoscopy with new biopsies. Early recognition of this clinical entity is of paramount importance to implement appropriate therapy (conservative or surgical) and avoid potentially fatal treatment of presumed inflammatory or infectious bowel diseases.

## 1. Introduction

Ischemic colitis accounts for 1 in 1000 hospitalizations, but its incidence is underestimated because it usually has a mild and transient nature [1].

The clinical presentation is variable. Most cases of the nongangrenous form are transient and resolve spontaneously without complications. On the other hand, high morbidity and mortality and urgent operative intervention are the hallmarks of gangrenous ischemic colitis.

Rectal sparing is considered a basic diagnosing feature, which discriminates ischemic colitis from other colitis, since ischemic proctitis is a rare entity [2]. An average of 10–20% of cases are presented as a distal obstructing mass.

We present a severe case of ischemic proctitis that was mimicking a rectal cancer.

## 2. Case Presentation

An 80-year old male with end-stage heart failure, severe coronary disease, and mild renal failure presented to our hospital with haematochezia and left sided abdominal pain during the last twenty four hours. Physical examination demonstrated tachycardia, hypotension (BP: 95 mm Hg), and a slight tenderness of abdominal wall. The ECG did not reveal any arrhythmia. Rectal examination revealed a hard, irregular rectal mass 3-4 cm above the anal verge. 

The patient was on medication including digoxin, b-blockers, and low doses of aspirin. He had no history of weight loss, change of abdominal movements, or any other episode of bloody stools.

Laboratory tests showed anemia (hemoglobin: 7 mg/dL) with a normocytic normochromic peripheral blood smear, a mild leukocytosis (WBC: 12000), and a moderate increase of urea and creatinine. Patient was easily stabilized with transfusion (three units of RBC) and intravenous fluids cautiously given, and total colonoscopy was followed under cardiologist supervision. No analgesia was given.

**Figure 1:**
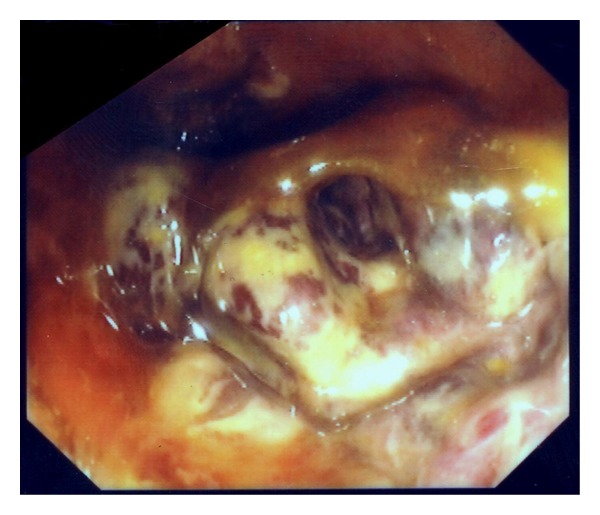
Two days after the admission. The lumen of the rectum was partially obstructed by a huge ulcerating rectal mass, 3-4 cm above the dental line.

The endoscopy revealed a huge ulcerating mass at distal rectum with an uneven, hemorrhagic surface causing partial obstruction of the lumen (Figure 1). The mucosa of upper rectum and sigmoid colon was erythematous and edematous with superficial ulcerations and small submucosal hemorrhagic nodules. No active bleeding was seen. 

Colonic ischemia of upper rectum and sigmoid colon was clearly suspected. Stool cultures were taken, and patient was given intravenously ciprofloxacin and metronidazole. Antiplatelet and vasoconstrictive drugs such digoxin and b-blockers were withdrawn. Patient's clinical and laboratory condition was steadily improved. 

Since the distal rectal mass had been mimicking a rectal cancer, the patient underwent an abdominal computer tomography of abdomen and pelvis which revealed a circumferential transmural thickening of a long segment of the distal colon without lymph node enlargement or liver metastasis. 

Serum CEA, Ca 19.9, and PSA were in normal range. Stool cultures were negative. Biopsies demonstrated mucosal and submucosal haemorrhage, edema, and superficial ulceration. 

**Figure 2:**
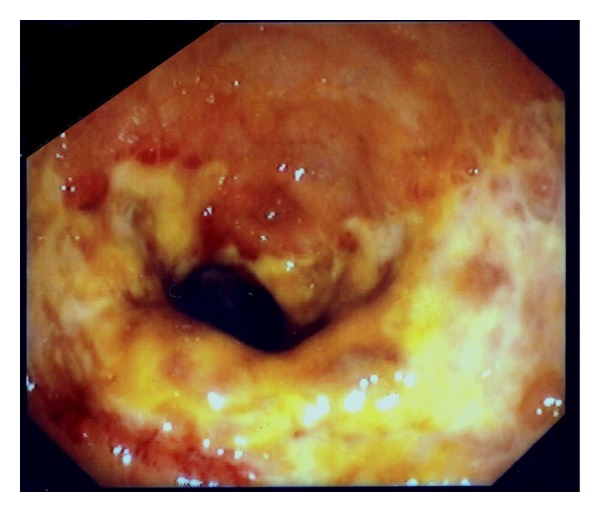
Two weeks after the treatment. Superficial coalescing ulcers were seen at the same site where the obstructive mass was initially observed.

The patient recovered completely. Two weeks later, proctoscopy showed a great improvement of endoscopic view with superficial coalescing ulcers at rectum (Figure 2). One month later mucosal healing was almost complete. Three years later the patient remains asymptomatic. 

## 3. Discussion

Acute ischemia of the rectum is rare (<6%) because of its extensive collateral blood supply. It is more often seen after aortoiliac surgery or as a result of compromised blood flow to the rectum from mesenteric vascular interventions albeit case reports after an epilepsy episode or anaphylactic shock have been reported [3–5].

The underlying mechanism leading to ischemic proctosigmoiditis is not completely understood. Thorén et al. [6] observed that hypoperfusion due to hypovolemia, superimposed on atherosclerotic narrowing of the aortoiliac vessels, may contribute to the pathogenesis of proctosigmoid ischemia. Risk factors include major vascular occlusive disease, disruption of collateral circulation, and low perfusion state. 

According to Sharif and Hyser [7] aortoiliac occlusive disease accounted for nearly half of the cases, while 40% was secondary to a low perfusion state. At the same retrospective study, mortality was very high (40%) when transmural necrosis of the rectal wall was encounterd. 

Treatment is nonoperative for nongangrenous ischemic proctocolitis (80–90%), whereas surgery is necessary for gangrenous, transmural rectal ischemia. Flobert and colleagues [8] evaluated the predisposing factors associated with severe outcome in patients with ischemic colitis. They found that chronic renal failure, hemodialysis, short delay between symptoms and diagnosis, and mainly right side involvement were significantly associated with unfavorable outcome [8, 9]. Chung et al. [10] developed a novel prognostic scoring model supported that vital instability at admission and endoscopic ulceration are negative prognostic factors. Añón et al. [11] noticed that seriously ill patients had less hematochezia than slightly ill patients although they had more tachycardia and lower levels of hemoglobin. According to Antolovic et al. [12] the mortality of patients requiring surgery for ischemic colitis will remain high as the majority of afflicted patients are patients with significant comorbidities in a reduced general condition. 

An average of 10%–20% of patients with ischemic colitis usually have a distal potentially obstructing lesion or disorder such as cancer, diverticulitis, or faecal impaction. When mucosal and submucosal edema is severe and hemorrhagic nodules are large enough, the lesion could mimic a neoplasm. Such cases have been well described [13–15]. The situation could be more complicated when ischemic proctitis resembles an ulcerating vegetating mass, because rectal involvement is one of the major endoscopic exclusion criteria of the diagnosis of ischemia. Another diagnostic and therapeutic dilemma is represented by the distal location of the lesion and the ulcer craters formations. 

The findings of CT scan are often nonspecific and misleading. CT scan may suggest the diagnosis and may identify other causes but cannot determine the severity of the condition. 

Colonoscopy represents the gold standard in diagnosing and determining the extension of the ischemic lesion [16]. Histological examination can offer a diagnosis for colitis, but it cannot exclude the diagnosis of cancer. 

The treatment of choice is conservative, initially with a close clinical followup, and after the stabilization, a repetition of rectal endoscopy with new biopsies would be mandatory. In our case, transmural involvement and patient's comorbidities were negative prognostic factors indicating an urgent operative intervention. 

Early recognition of this clinical entity is of paramount importance to implement appropriate therapy (conservative or surgical) and avoid potentially fatal treatment of presumed inflammatory or infectious bowel diseases. 

As survival expectancy is steadily increased, gastroenterologists should be aware of this rare entity which in its severe type can be both a clinical and therapeutic dilemma. 

## References

[1] Theodoropoulou A, Koutroubakis IE (2008). Ischemic colitis: clinical practice in diagnosis and treatment. *World Journal of Gastroenterology*.

[2] Bharucha AE, Tremaine WJ, Daniel Johnson C, Batts KP (1996). Ischemic proctosigmoiditis. *American Journal of Gastroenterology*.

[3] Abhishek K, Kaushik S, Kazemi MM, El-Dika S (2008). An unusual case of hematochezia: acute ischemic proctosigmoiditis. *Journal of General Internal Medicine*.

[4] Arya N, Hawe MJG, Ozo C (2004). Diclofenac suppositories and acute ischaemic proctitis. *Ulster Medical Journal*.

[5] Park S, Chun HJ, Keum B (2010). Anaphylactic shock-induced ischemic proctocolitis following bee stings: first case report. *Endoscopy*.

[6] Thorén A, Ricksten S, Lundin S, Gazelius B, Elam M (1998). Baroreceptor-mediated reduction of jejunal mucosal perfusion, evaluated with endoluminal Laser Doppler flowmetry in conscious humans. *Journal of the Autonomic Nervous System*.

[7] Sharif S, Hyser M (2006). Ischemic proctitis: case series and literature review. *American Surgeon*.

[8] Flobert C, Cellier C, Berger A (2000). Right colonic involvement is associated with severe forms of ischemic colitis and occurs frequently in patients with chronic renal failure requiring hemodialysis. *American Journal of Gastroenterology*.

[9] Sotiriadis J, Brandt LJ, Behin DS, Southern WN (2007). Ischemic colitis has a worse prognosis when isolated to the right side of the colon. *American Journal of Gastroenterology*.

[10] Chung JW, Cheon JH, Park JJ, Jung ES, Choi EH, Kim H (2010). Development and validation of a novel prognostic scoring model for ischemic colitis. *Diseases of the Colon and Rectum*.

[11] Añón R, Boscá MM, Sanchiz V (2006). Factors predicting poor prognosis in ischemic colitis. *World Journal of Gastroenterology*.

[12] Antolovic D, Koch M, Hinz U (2008). Ischemic colitis-analysis of risk factors for postoperative mortality. *Langenbeck’s Archives of Surgery*.

[13] Deepak P, Devi R (2011). Ischemic colitis masquerading as colonic tumor: case report with review of literature. *World Journal of Gastroenterology*.

[14] Feldman M, Friedman L, Sleisenger M (2002). Intestinal ischemia. *Sleisinger & Fordtran's Gastrointestinal & Liver Disease. Pathophysiology, Diagnosis, Management*.

[15] Lee HH, Agha FP, Owyang C (1986). Ischemic colitis masquerading as colonic tumor: an unusual endoscopic presentation. *Endoscopy*.

[16] Wiesner W, Mortelé KJ, Glick man JN, Ji H, Khurana B, Ros PR (2002). CT findings in isolated ischemic proctosigmoiditis. *European Radiology*.

